# Stakeholders’ Experience of Postpartum Psychosis Recovery in UK Mother and Baby Units: A Systematic Review and Conceptual Framework

**DOI:** 10.1192/bjo.2024.114

**Published:** 2024-08-01

**Authors:** Jillian Barry

**Affiliations:** North London Partnership, London, United Kingdom. HARP Pre-doctoral Research Programme, London, United Kingdom. Queen Mary University, London, United Kingdom

## Abstract

**Aims:**

- Identify themes in experience of Postpartum Psychosis (PP) recovery in Mother and Baby Units (MBUs) from the perspective of mothers, partners and MBU professionals.
- Develop a Conceptual Framework of recovery from PP in the MBU setting.

**Methods:**

Systematic review using published and unpublished literature identified through database searches and grey literature sources. A narrative synthesis approach was taken and used to form a Conceptual Framework of recovery from PP in the MBU setting.

**Results:**

Four databases were searched, yielding 8 includable studies. A further 3 grey literature sources met the inclusion criteria. Most of the sources focussed on the womens' experience of recovery.

Stakeholders experienced MBUs as providing a positive therapeutic milieu for recovery. The broad themes identified for improvement encompassed: knowledge of PP, accessibility of services and discharge practises.

**Conclusion:**

This review provides valuable insights into the experience of recovery from PP within UK MBUs from the perspectives of multiple stakeholders. Areas for improvement identified include antenatal education on PP, knowledge of PP amongst non-specialist healthcare professionals, partner involvement in care, and discharge processes.

The outcomes of this review have the potential to shape the design, implementation, and expansion of MBUs and their practices both nationally and internationally.

